# Description of *Barathricolathermophilus*, a new species from a deep-sea hydrothermal vent field in the Indian Ocean with redescription of the *Barathricola* type species (Crustacea, Copepoda, Cyclopoida)

**DOI:** 10.3897/zookeys.865.35827

**Published:** 2019-07-22

**Authors:** Viatcheslav N. Ivanenko, Jimin Lee, Cheon Young Chang, Il-Hoi Kim

**Affiliations:** 1 Department of Invertebrate Zoology, Biological Faculty, Lomonosov Moscow State University, Leninskie Gory, 1-12, Moscow 119992, Russia Lomonosov Moscow State University Moscow Russia; 2 Marine Ecosystem Research Center, Korea Institute of Ocean Science & Technology, 385 Haeyang-ro, Yongdo-gu, Busan 49111, South Korea Marine Ecosystem Research Center, Korea Institute of Ocean Science & Technology Busan South Korea; 3 Department of Biological Science, Daegu University, 201 Daegudae-ro, Gyeongsan 38453, South Korea Daegu University Gyeongsan South Korea; 4 Korea Institute of Coastal Ecology, 397 Seokcheon-ro, Bucheon 14449, South Korea Korea Institute of Coastal Ecology Bucheon South Korea

**Keywords:** Central Indian Ridge, key, Onnuri vent field, Schminkepinellidae, taxonomy

## Abstract

Re-study of the type species of the genus *Barathricola* Humes, 1999 (Copepoda, Cyclopoida, Schminkepinellidae) described from the Pacific Ocean (Juan de Fuca Ridge), and study of the species *Barathricolathermophilus***sp. nov.** from a deep-sea hydrothermal vent field on the Central Ridge in the Indian Ocean revealed a derived feature and widespread geographic distribution of this deep-sea genus of cyclopoids. The derived feature of *Barathricola* is the sexually dimorphic third endopodal segment of leg 3 possessing a small outer terminal spine together with spine-like outgrowths on this segment. The new species differs from *Barathricolarimensis* Humes, 1999 in not expressing sexual dimorphism in leg 5, having three spines and one seta on its exopod in both sexes (*B.rimensis* has three spines and one seta on the female exopod but three spines and two setae on the male exopod) and in having broader caudal rami which are 8.9 times longer than wide in the female (this ratio for *B.rimensis* is 11). An amended diagnosis of the genus *Barathricola*, a key and a table of morphological differences for all species of Schminkepinellidae are given.

## Introduction

Cyclopoids of the family Schminkepinellidae were discovered in the deep-sea and in marine caves (Martínez Arbizu 2006). The genera initially allocated to Schminkepinellidae were the monotypic genera *Cyclopinella* G.O. Sars, 1913 *Barathricola* Humes, 1999, *Einslepinella* Martínez Arbizu, 2006, *Muceddina* Jaume & Boxshall, 1996, and *Schminkepinella* Martínez Arbizu, 2006. The type species *Cyclopinellatumidula* Sars, 1913 was collected from benthic muds off the Norwegian coast (Sars 1913). *Muceddinamultispinosa* Jaume & Boxshall, 1996, the only species of this genus, was collected from anchialine caves on Mediterranean and eastern Atlantic islands (Jaume and Boxshall 1996). Humes (1999) recorded *Barathricolarimensis* Humes, 1999 from a depth of 2254 m at a hydrothermal vent area in the northeastern Pacific. Martínez Arbizu (2006) described *Schminkepinellaplumifera* from a depth of 3211 m and *Einslepinellaulrichi* from a depth of 529 m in the Arctic Ocean, both as new genera and species. The family was considered as the sister group of Poecilostomatoida allocated to the order Cyclopoida (Martínez Arbizu 2000). A molecular analysis conducted by Khodami et al. (2017) placed Schminkepinellidae as the sister group of Poecilostomatoida but was not verified by the analysis of Mikhailov and Ivanenko (2019) based on data provided by the authors. Karanovic (2008) described shallow water *Cyclopinellatincanbayensis* Karanovic, 2008 from Queensland in Australia, synonymised *Barathricola* and *Muceddina* with *Cyclopinella* based on characters shared by these two genera and *Cyclopinella*.

In June 2018 the Korea Institute of Ocean Science and Technology (KIOST) made an expedition to deep-sea hydrothermal vent fields on the Central Indian Ridge in the Indian Ocean and sampled benthic habitats, using the research vessel ISABU. Several species of copepods were discovered from this expedition. A new species of the genus *Barathricola*, which is described herein, is among these copepods. In addition, to verify diagnostic characters and the validity of the genus *Barathricola* we restudied the type specimens of the genera *Barathricola* and *Muceddina*.

## Materials and methods

Samples of the meiobenthos around the hydrothermal vents of the Onnuri Vent Field (OVF), Central Indian Ridge, Indian Ocean, were made using a TV-grab (Video-Guided Hydraulic Grab, Octopus, Germany) during the deep-sea expedition of the research vessel RV ISABU of the KIOST in June 2018. Sampled sediments were fixed and preserved in 10% formalin for a couple of months. Copepods were sorted out from the sediments and stored in 80% ethanol.

Prior to description of the species, selected copepod specimens were soaked in lactic acid. Dissections were performed using the reversed slide method of Humes and Gooding (1964). The specimens of *Barathricola* and *Muceddina* were studied with a Leica DMR compound microscope using bright-field and differential interference contrast optics. Drawings were made with a camera lucida mounted on the microscope. In the description, the body lengths of the specimens were measured from the anterior margin of the cephalothorax to the end of caudal rami, excluding setae. Type specimens of the new species have been deposited in the Marine Biodiversity Institute of Korea (MABIK), Seocheon, Korea.

## Systematic account

### Order Cyclopoida Burmeister, 1834

#### Family Schminkepinellidae Martínez Arbizu, 2006

##### Genus Barathricola

Taxon classificationAnimaliaCyclopoidaSchminkepinellidae

Humes, 1999

5d4b0d34-ad7e-48dd-9ddf-3f6ad0f3a5b1

###### Amended diagnosis.

Cyclopoida. Prosome slender, 5-segmented. Urosome 5-segmented in female, 6-segmented in male, first somite with leg 5. Caudal rami elongate, bearing six or seven setae. Antennule 14-segmented in female and 17-segmented in male; geniculation of male antennules between segments 15 and 16. Antenna 4-segmented, without exopod; armature formula 0-1-5-7. Mandible palp biramous, with elongate basis; endopod 2-segmented, first segment with two, second segment with four setae; exopod small, indistinctly 2-3-segmented, with two terminal setae. Maxillulary coxal endite absent. Maxilla with praecoxa, coxa, basis, and 3-segmented endopod armed with four, two and four setae, respectively. Maxilliped 7-segmented, with syncoxa bearing three (1+2) setae, basis with two setae and 5-segmented endopod with setal formula 1, 1, 1, 1, 3. Legs 1-4 biramous, with 3-segmented rami; armature formula as in Table 1. Leg 1: inner margin of basis bearing long flattened setules. Third endopodal segment of leg 3 with three spines and three setae (1,II,I+2); in male with small outer terminal spine near spine-like outgrowth. Middle endopodal segment of leg 4 with distal inner seta modified into spine. Leg 5 consisting of coxa, basis, and exopod, with intercoxal sclerite; endopod absent; setal formula -0; 1-0; I, I+1+I in female and 0-0; 1-0; I, I+1+I or 0-0; 1-0; I, I+1+I, 1 in male.

**Table 1.** Spine and setal formulae of legs 1-4 in *Barathricolarimensis* Humes, 1999. Roman numerals indicate spines, and Arabic numerals setae.

	Coxa	Basis	Endopod	Exopod
Leg 1	0-1	1-I	0-1;0-1;1,2,3	I-0;I-1;III,I,4
Leg 2	0-1	1-0	0-1;0-2;1,2,3	I-0;I-1;III,I,5
Leg 3	0-1	1-0	0-1;0-2;1,II,I+2	I-0;I-1;III,I,5
Leg 4	0-1	1-0	0-1;0-1+I;I,II,II	I-0;I-1;II,I,5

###### Type species.

*Barathricolarimensis* Humes, 1999. *Barathricolathermophilus* sp.nov. is the second species of this genus.

##### Barathricola thermophilus sp. nov.

Taxon classificationAnimaliaCyclopoidaSchminkepinellidae

0596004c-bc1d-4091-ad96-0cac3f3d6518

http://zoobank.org/3AE79CB6-053D-406B-ADA5-69477A4D462A

Figs 1 , 2 , 3

###### Type locality.

The hydrothermal vent field of OVF (11°24'52.97"S, 66°25'25.48"E) on the Central Indian Ridge in the Indian Ocean; sediments at 2022 m in depth.

###### Type material.

Holotype (♀, MABIK CR00244723) and paratypes (6 ♀♀, 6 ♂♂, MABIK CR00244724) have been deposited in the MABIK. Dissected paratypes (2 ♀♀, 1 ♂) are retained in the collection of the last author. All type specimens collected on 23 June 2018 from the type locality.

###### Description of female.

**Figure 1.**
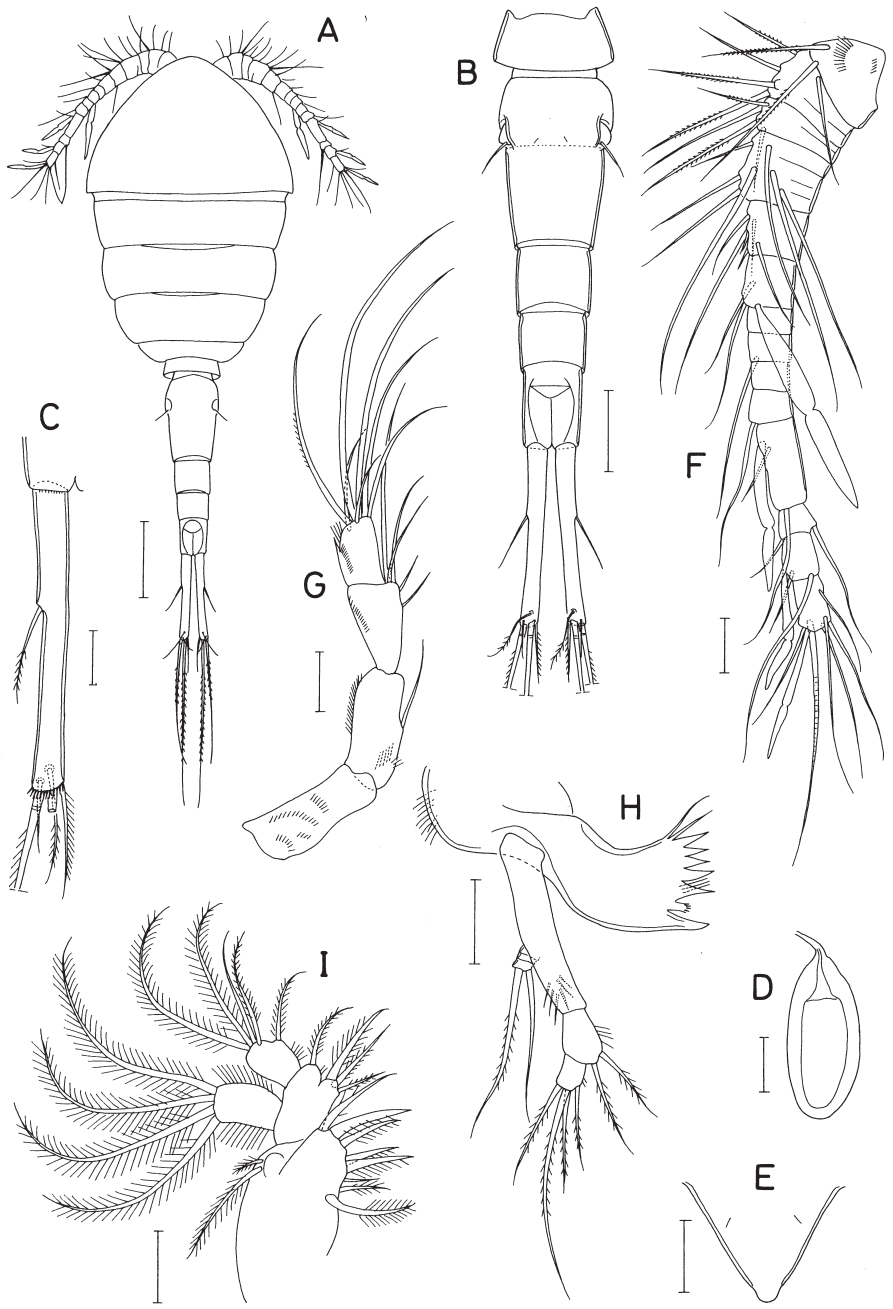
*Barathricolathermophilus* sp. nov., female: **A** habitus, dorsal **B** urosome, dorsal **C** right caudal ramus, ventral **D** spermatophore **E** rostrum **F** antennule **G** antenna **H** mandible **I** maxillule. Scale bars: 0.1 mm (**A**), 0.05 mm (**B**), 0.02 mm (**C–I**).

Body (Fig. 1A) slender. Length of dissected and described specimen 776 μm. Other three measured specimens 700, 710, and 715 μm, respectively. Prosome nearly oval, 400 μm long, slightly longer than urosome, consisting of cephalosome and four pedigerous somites. Greatest width of prosome 273 μm across cephalosome. Cephalosome with angular posterolateral corners. First to third pedigerous somites almost equal in length. Fourth pedigerous somite distinctly shorter and narrower than the third. Urosome (Fig. 1B) slender, 5-segmented. Fifth pedigerous somite 38 × 74 μm, broadened distally, with angular posterolateral corners. Genital double-somite 109 × 70 μm, 1.56 times as long as wide, gradually narrowing posteriorly; genital aperture located dorsolaterally at 38% region of double-somite length. Three free abdominal somites 40 × 47, 30 × 42, and 50 × 37 μm, respectively. Anal somite with large anal region and minute spinules along ventrodistal margin. Caudal ramus (Fig. 1C) 116 × 13 μm, 8.92 times as long as wide, more than twice as long as anal somite, armed with six setae and ornamented with row of spinules along ventrodistal margin; outer lateral seta located at 39% region of ramus length; spermatophore (Fig. 1D) attached to female 60 × 27 μm, with thick wall.

Rostrum (Fig. 1E) triangular, with thin-walled lobate distal apex. Antennule (Fig. 1F) 225 μm long, longer than cephalosome, and 14-segmented. Eleventh segment the longest. Armature formula 2-5-4-7-6-(2 + aesthetasc)-0-1-0-1-(2 + aesthetasc)-2-(2 + aesthetasc)-(6 + aesthetasc). Second and third segments each with a trace of one subdivision, and fourth segment with three subdivisions. First segment with two rows of fine spinules. Most of setae naked, except several feebly pinnate ones of proximal two segments. Aesthetascs broad, constricted at region slightly distal to middle, and attenuated distally.

Antenna (Fig. 1G) 4-segmented, consisting of basis and 3-segmented endopod. Basis unarmed, ornamented with several rows of minute spinules. First endopodal segment 36 × 17 μm, with one seta on inner margin and minute spinules proximally and on outer margin. Second endopodal segment narrow proximally and gradually broadened distally, 30 × 18 μm, armed with five setae (three distal and two smaller subdistal) and ornamented with row of minute setules on outer side. Third endopodal segment 23 × 14 μm, armed with seven unequal setae distally, and ornamented with setules on outer side.

Labrum weak, easily destroyed. Mandible (Fig. 1H) consisting of coxa, basis, exopod, and endopod. Coxa with setules on outer margin; cutting margin of gnathobase with six acutely pointed teeth, two thin proximal setae, three setules between distal second and third teeth, and one small, transparent digitiform process bearing fine spinules distally between distal first and second teeth. Basis elongate, 42 × 9 μm, bearing five or six setules subdistally. Exopod small, indistinctly 3-segmented, armed only with two setae on third segment, outer one of these setae sparsely pinnate and slightly longer than inner one. Endopod 2-segmented, armed with two and four setae on first and second segments, respectively, all six setae sparsely pinnate; first segment with several setules on medial margin.

Maxillule (Fig. 1I) with eight setae on praecoxal arthrite; second distal seta spiniform. Coxal endite absent. Epipodite with two unequal setae. Basis with four setae, three proximal and one distal. Exopod with four large setae distally; setae becoming longer from outer to inner margin. Endopod shorter than exopod, armed with five setae, one on medial margin, and four distally.

**Figure 2.**
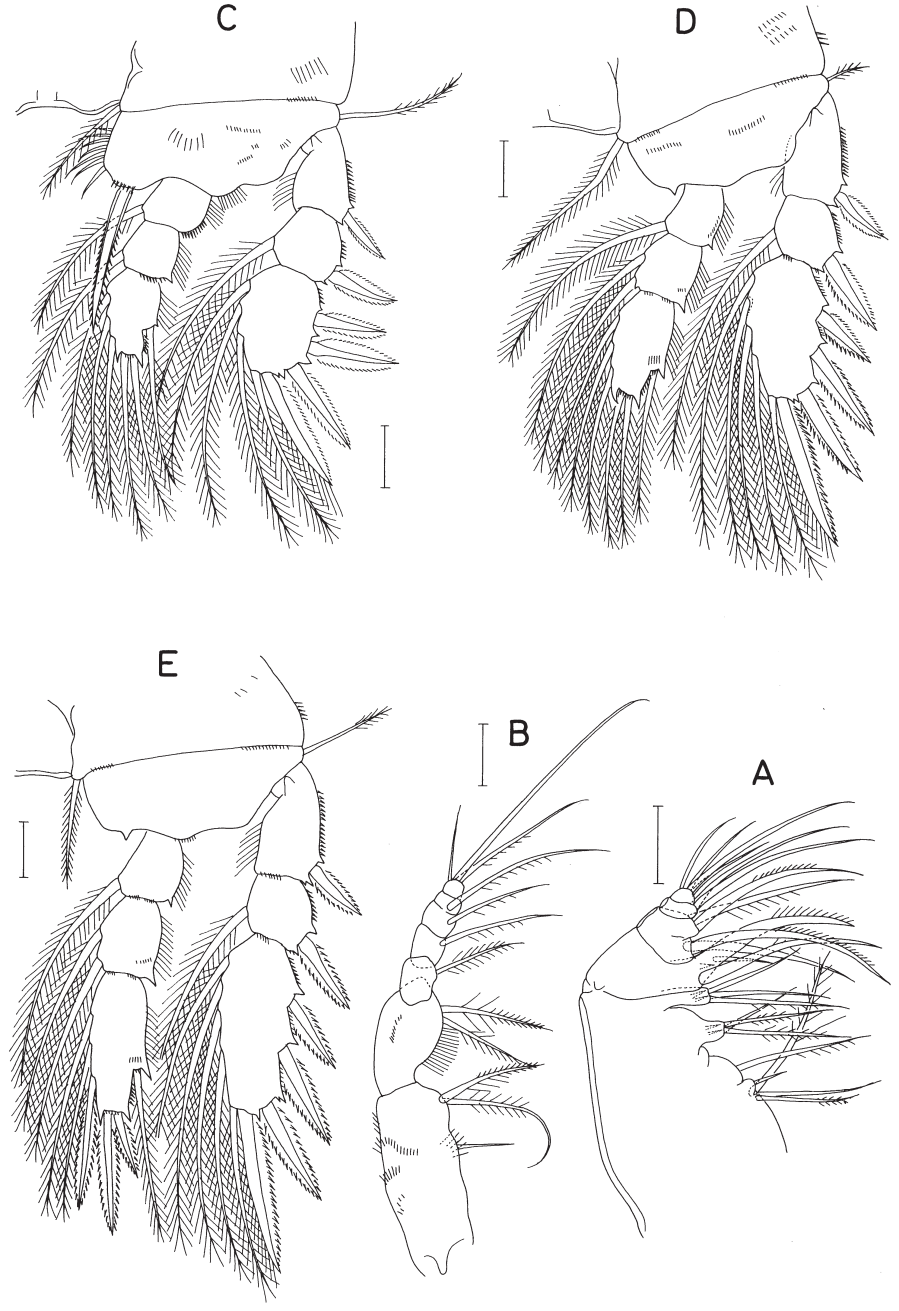
*Barathricolathermophilus* sp. nov., female: **A** maxilla **B** maxilliped **C** leg 1 **D** leg 2 **E** leg 3. Scale bars: 0.02 mm.

Maxilla (Fig. 2A) stout, 5-segmented, consisting of syncoxa, basis, and 3-segmented endopod. Syncoxa armed with 11 setae, grouped as four, one, three, and three on first to fourth endites, respectively; third and fourth endites ornamented with two spinules at distal region. Basis armed with three unequal setae (one large, proximally unarticulated, spiniform, one long, and one small setae) and ornamented with one spinule. First endopodal segment with four setae (two proximal and two distal). Second endopodal segment with two long setae; third endopodal segment small, with four setae (one long and three shorter).

Maxilliped (Fig. 2B) slender, 7-segmented, consisting of syncoxa, basis, and 5-segmented endopod. Syncoxa with several scattered rows of minute setules, and armed with three setae, proximal one small and naked. Basis with two setae and rather long setules on medial margin. Endopod armed with one, one, one, one, and three setae on first to fifth segments, respectively; middle seta on terminal segment naked, much longer than other setae, longer than basis and endopod combined. Articulation incomplete between third and fourth endopodal segments.

**Figure 3.**
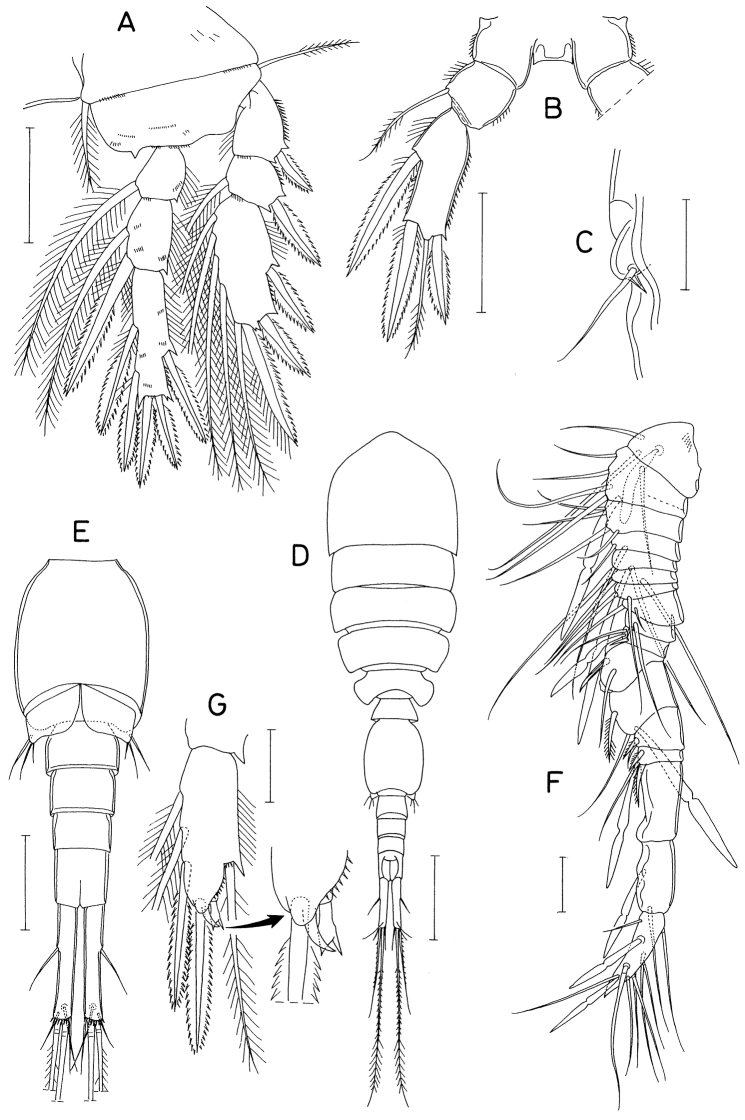
*Barathricolathermophilus* sp. nov. female: **A** leg 4 **B** leg 5 **C** left genital aperture. Male: **D** habitus, dorsal **E** genital somite and abdomen, ventral **F** antennule **G** third endopodal segment of leg 3. Scale bars: 0.05 mm (**A, B, E**), 0.02 mm (**C, F, G**), 0.1 mm (**D**).

Legs 1–4 (Figs 2C–E; 3A) with 3-segmented exopod and endopod, lacking inner seta on first exopodal segment; third exopodal segment distinctly broader than proximal segments. All intercoxal sclerites smooth without spinule/setule array along distal margin and on both anterior and posterior surfaces. Endopods of legs 1-3 shorter than exopod, but that of leg 4 distinctly longer than exopod. Leg 1 (Fig. 2C) basis with seven thick setules on inner margin; inner distal spine large, 48 μm long, extending to middle of third endopodal segment, spinulose along both margins. Leg 2 (Fig. 2D) with inner coxal seta characteristically bent at proximal quarter; outer seta on basis shorter than those of legs 1, 3 and 4. Inner distal corner of basis of legs 2-4 with pointed dentiform process. Leg 3 (Fig. 2E) with two distal spines on third endopodal segment (outer spine ca. half as long as inner spine). Leg 4 (Fig. 3A), third endopodal segment elongate, 3.6 times as long as wide; inner distal seta on second endopodal segment and two inner and one outer setae on third endopodal segment transformed to spines. Armature formula for legs 1-4 as in Table 1.

Leg 5 (Fig. 3B) 3-segmented, consisting of coxa, basis and exopod; intercoxal sclerite small, narrow, with pointed outer distal corners and slightly concave distal margin. Coxa quadrate, unarmed, not articulated from somite. Basis also quadrate, armed with one pinnate seta outer distally. Exopod 54 × 24 μm, 2.25 times as long as wide, armed with three spines (two distal and one outer) and one pinnate seta; medial margin spinulose and outer margin setulose.

Leg 6 (Fig. 3C) represented by one spinule and one naked seta on genital operculum.

###### Description of male.

Body (Fig. 3D) much narrower than that of female, 582 μm long. Prosome 314 × 153 μm, approximately twice as long as wide. First pedigerous somite slightly narrower than cephalosome and second pedigerous somite. Urosome 6-segmented. Fifth pedigerous somite narrower than genital somite. Genital somite (Fig. 3E) 86 × 72 μm, longer than wide, with well-developed genital operculum. Four abdominal somites 25 × 40, 23 × 34, 20 × 31, and 30 × 28 μm, respectively. Caudal ramus 6.1 times as long as wide (61 × 10 μm); arrangement and locations of caudal setae as in female.

Rostrum as in female. Antennule (Fig. 3F) 17-segmented; armature formula (2 + aesthetasc)-(5 + aesthetasc)-4-2-(2 + aesthetasc)-2-2-2-2(2 + aesthetasc)-(1 + spine)-(2 + aesthetasc)-2-[3 + aesthetasc (or 2+aesthetasc)]-[0 (or 1)]-(1 + aesthetasc)-(9 + 2 aesthetascs); eleventh segment with short posterior margin and much longer anterior margin, spine on this segment slender. Antenna as in female.

Mandible and other mouth appendages as in female.

Legs 1, 2, and 4 also as in female. Leg 3 sexually dimorphic; third endopodal segment (Fig. 3G) bearing two spines, three setae, and distally two small specialized elements, one curved, non-articulating, spinule-like element and one straight, distally bifurcate articulating element.

Leg 5 as in female. Leg 6 (Fig. 3E) represented by three naked setae on genital operculum, medial one smaller than other two.

###### Etymology.

The specific name *thermophilus* is a combination of Greek words *therm* (=heat) and *phil* (=loving), referring to the finding of the new species in a hydrothermal vent field.

##### Barathricola rimensis

Taxon classificationAnimaliaCyclopoidaSchminkepinellidae

Humes, 1999

66938c11-6cfd-45da-8590-917140e0b2d7

Figs 4 , 5 , 6 , 7

###### Material.

Females and males from the type locality dissected by A.G. Humes and marked as *Barathricolarimensis* in the Zoological Museum of Lomonosov Moscow State University (collection numbers: w.cyc.sch.1.1-1.5). The hydrothermal vent field is at Juan de Fuca Ridge (44°08.6'N, 129°42'W) in the northeastern Pacific, 26 August 1996 at 2254 m depth.

###### Redescription of female.

Body as in original description. Differs from *Barathricolathermophilus* sp. nov. in following features.

**Figure 4.**
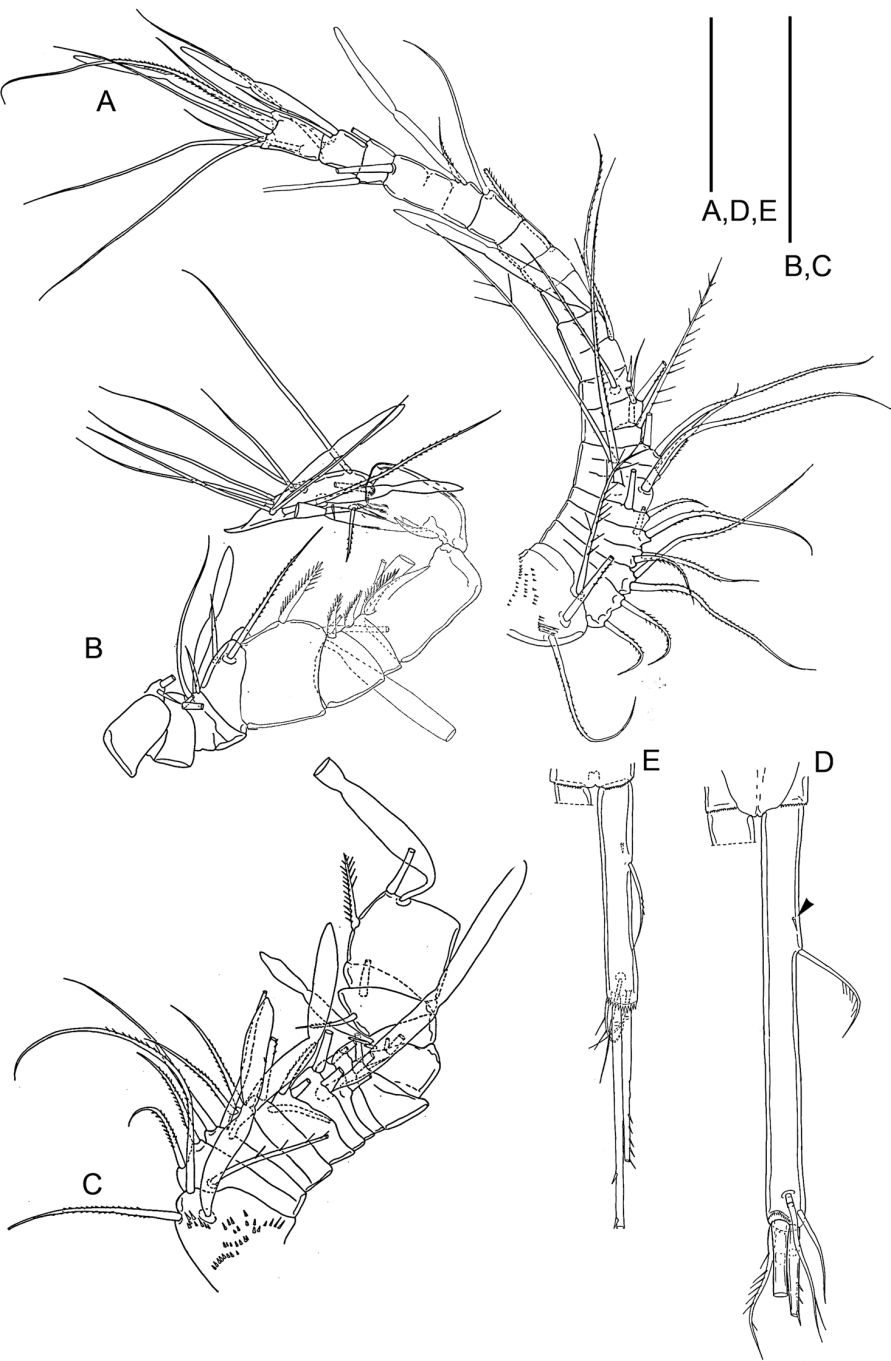
*Barathricolarimensis* Humes, 1999: **A** antennule of female **B** antennule of male, distal segments 8-17 **C** antennule of male, proximal segments 1-12 **D** caudal ramus of female **E** caudal ramus of male. Scale bars: 0.05 mm.

Caudal ramus (Fig. 4D) elongate, 99 × 9 mm, ratio of length to width 11:1. Outer lateral seta located approximately at junction of first and second thirds of ramus. Dorsal seta short. Outermost terminal seta also short, placed dorsally. Innermost terminal seta short. All these setae smooth. Two long median terminal setae 117 mm (outer) and 234 mm (inner), both with lateral setules. Few minute spinules at distal outer corner of ramus.

Antennule (Fig. 4A) 14-segmented with numerous subdivisions. Armature formula: 3-8-8-5-3-0-1-0-1-(2 + aesthetasc)-(2 + aesthetasc)-(2 + aesthetasc)-(6 + aesthetasc).

**Figure 5.**
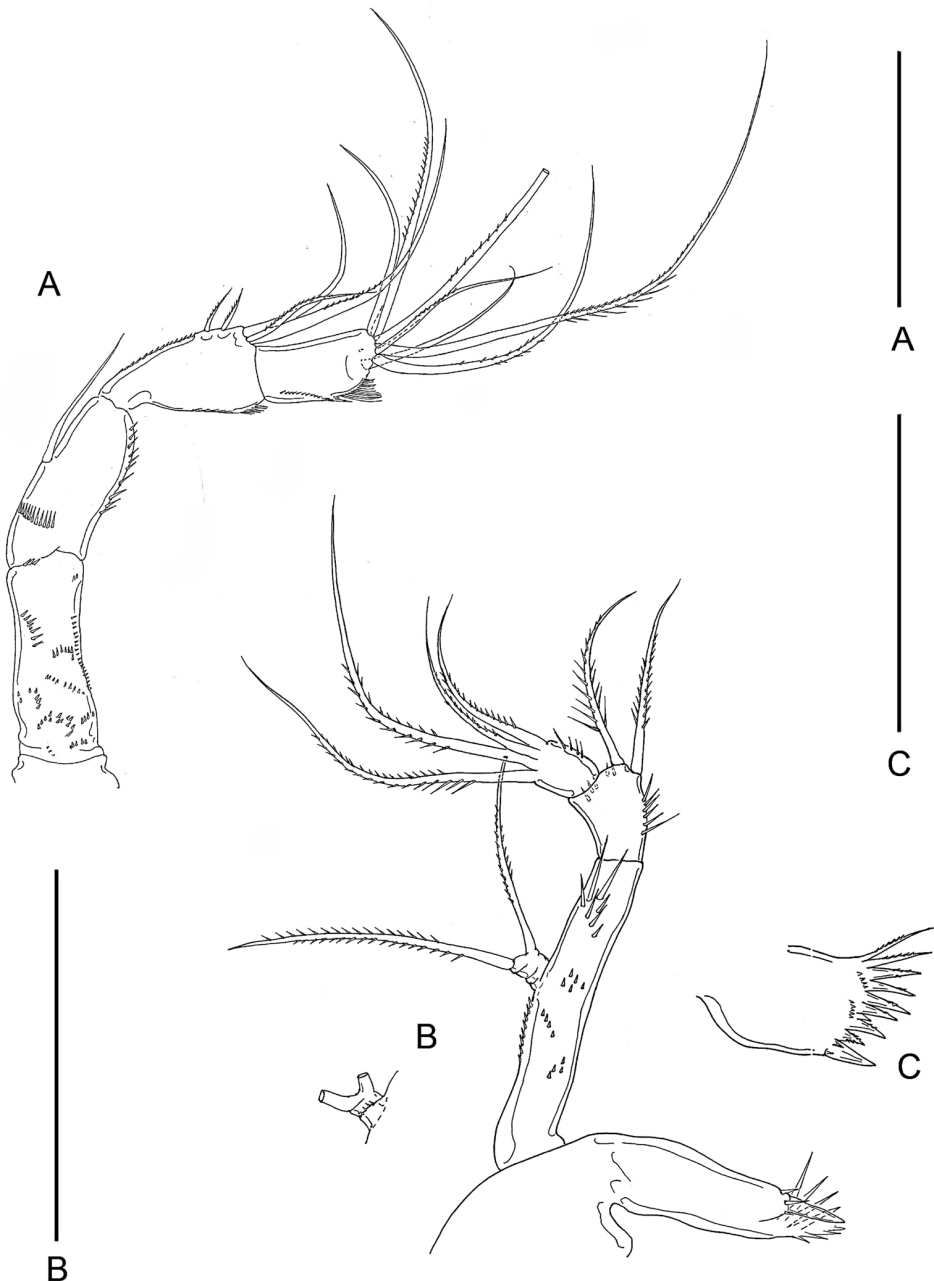
*Barathricolarimensis* Humes, 1999, female: **A** antenna **B** mandible **C** distal part of the mandibular gnathobase. Scale bars: 0.05 mm.

Antenna (Fig. 5A) four-segmented, with coxa, basis, and two-segmented endopod, armed with 0, 1, 5, and 7 setae. Exopod absent. Length 122 mm without setae.

Mandible (Fig. 5B, C) with coxa having medially directed gnathobase armed distally with row of seven or eight slender teeth. Palp biramous. Basis elongate, with minute exopodal process carrying two long setae, and two prominent setae distally on margin of basis; endopod two-segmented, first segment small, trapezoidal, bearing two setae and row of minute spinules, second segment small with four distal setae and row of minute spinules along anterior edge.

**Figure 6.**
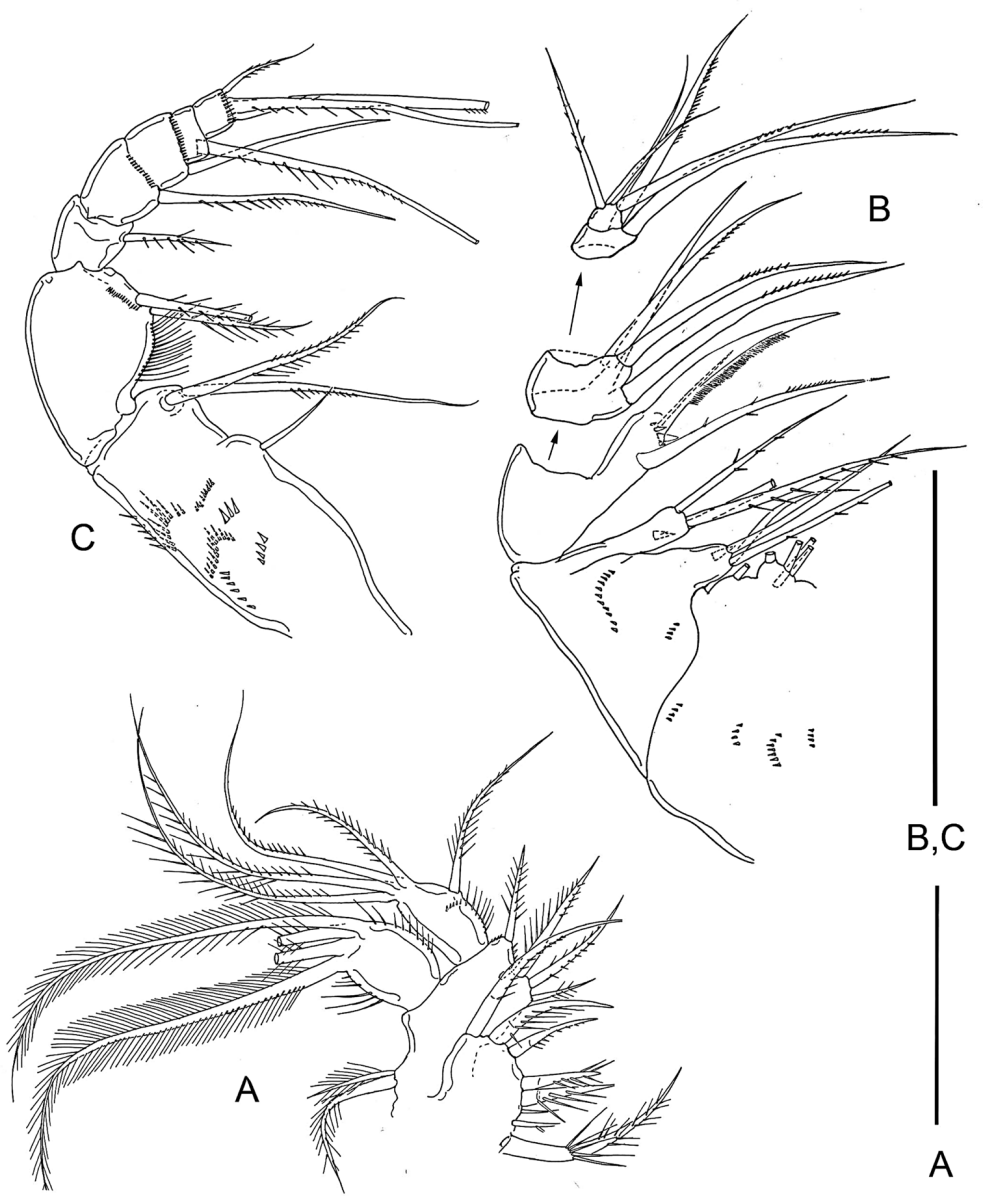
*Barathricolarimensis* Humes, 1999, female: **A** maxillule **B** maxilla **C** maxilliped. Scale bars: 0.05 mm.

Maxillule (Fig. 6A) with large praecoxa bearing arthrite with eight setae; coxa-basis with 3+1 setae; exopod with two short stout setae and two long slender setae; endopod with five setae.

Maxilla (Fig. 6B) with praecoxa having two endites, proximal endite bearing four setae, distal endite represented by single seta. Coxa with two endites, both with three setae. Basis with endite bearing three setae, one short, one long and slender, and one stout and claw-like, and having few minute subterminal spinules. Endopod three-segmented, with first segment having two endites with two setae each, small second segment bearing two setae, and minute third segment with four setae.

Maxilliped (Fig. 6C) with both coxa and basis swollen medially and bearing three and two setae, respectively; endopod slender, consisting of five segments armed with 1, 1, 1, 1, and 3 setae. Coxae of maxillipeds joined ventrally by one sclerotized line.

**Figure 7.**
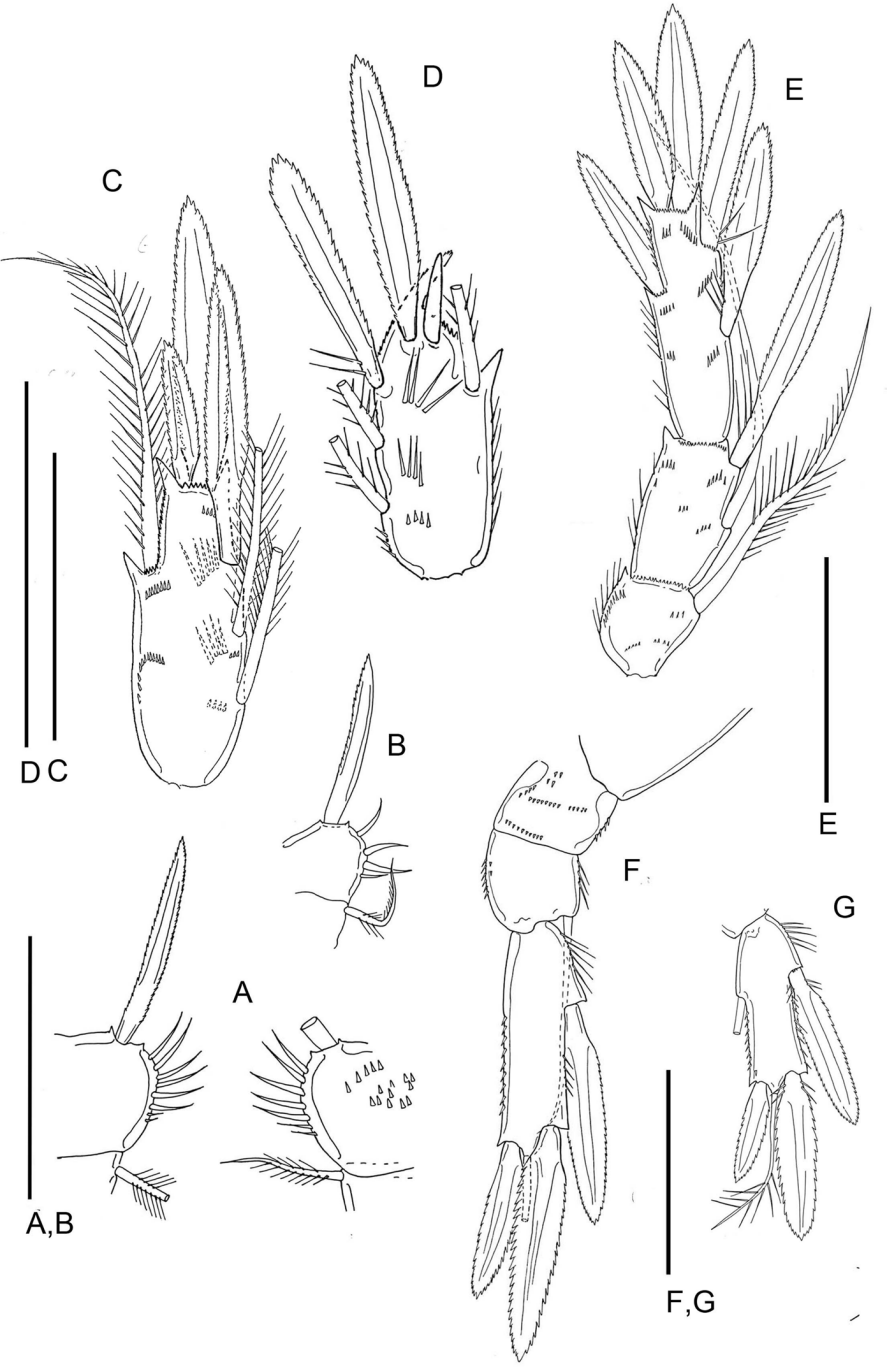
*Barathricolarimensis* Humes, 1999: **A** leg 1 of female, inner part of protopod **B** leg 1 of male, inner part of protopod **C** leg 3 of female, distal endopodal segment, posterior **D** leg 3 of male, distal endopodal segment, anterior **E** leg 4 of female, endopod, anterior **F** leg 5 of female, exopod, anterior **G** leg 5 of male, distal segment. Scale bars: 0.05 mm.

Legs 1–4 (Fig. 7A, C, E) biramous with three-segmented rami; armature formula for legs 1-4 as in Table 1. Leg 1 (Fig. 7A), inner side of basis with barbed spine and row of eight slender curved setules. Leg 3 (Fig. 7C) with 2 distal spines on third endopodal segment.

Leg 5 (Fig. 7F). Both legs connected by small quadrangular intercoxal sclerite and consisting of coxa, basis, and one-segmented exopod. Coxa and basis with setules along both sides. Basis with outer seta 44 mm long. Exopod 21 mm in greatest dimensions (15.5 mm wide distally) bearing three spines and one seta. Outer marginal barbed spine 57 mm, two terminal spines 58 mm (outer) and 41 mm (inner), both with minute outer spinules and longer inner fringelike setules. Seta between these two spines smooth, 55 mm. Outer margin of segment proximal to spine with setules; distal to spine and along inner side of segment with shorter setules, inner margin with minute spinules.

###### Redescription of male.

Differs from *Barathricolathermophilus* sp. nov. in following features:

Caudal ramus (Fig. 4E) resembling that of female but shorter, ratio 8.5:1. Antennule (Fig. 4B, C) 17-segmented; armature formula (2 + aesthetasc)-(5 + aesthetasc)-4-2-(2 + aesthetasc)-2-2-2-2(2 + aesthetasc)-(1 + spine)-(2 + aesthetasc)-2-[3 + aesthetasc (or 2+aesthetasc)]-[0 (or 1)]-(1 + aesthetasc)-(9 + 2 aesthetascs); eleventh segment with short posterior margin and much longer anterior margin, spine on this segment slender. Legs 1 (Fig. 7B) inner side of basis with barbed spine and row of eight slender curved setules. Leg 3 sexually dimorphic; third endopodal segment (Fig. 7D) bearing two spines, three setae, and distally two small specialized elements, one curved, non-articulating, spine-like element and one straight element. Leg 5 (Fig. 7G) different from that of female in having additional seta on inner margin of exopod (armature formula 0-0; 1-0; I, I+1+I, 1).

###### Remarks.

Martínez Arbizu (2006) established the family Schminkepinellidae into which he incorporated five genera, *Cyclopinella*, *Muceddina*, *Barathricola*, and his two new genera *Einslepinella* and *Schminkepinella*. The family is a monophyletic group of genera distinguished from other cyclopoid families by the reduction of a maxillulary coxal endite and the transformation of the distal inner seta on the middle endopodal segment of leg 3 into a spine (Martínez Arbizu 2006). None of the synapomorphies for the order Cyclopoida (a brush-like seta on the exopod of mandible, a brush-like seta on the exopod of maxillule, one or more flange-like setae on the endopod of swimming leg 4, pores with sensory dendrites laterally on the male cephalosome) proposed by Abiahy et al. (2006) are found in Schminkepinellidae. Karanovic (2008) described *Cyclopinellatincanbayensis* as a new species and synonymized two monotypic genera *Muceddina* and *Barathricola* with *Cyclopinella* and included these genera within Cyclopinidae based on the two major characters as synapomorphic shared by these nominal genera and *Cyclopinella*: the third endopodal segment of leg 4 with all armature elements transformed into spines and the three-segmented female leg 5 with an uniform armature and the elongate exopod. Karanovic (2008) recognized the mandibular palp as the most important morphological character differentiating species of *Cyclopinella* and its reduced segmentation and setation is consistent with reductions in other cephalic appendages and in the maxilliped. Our re-examination of the type species of the genus *Muceddina*, confirmed the original description and did not reveal the presence of a sexually dimorphic leg 3. This as well as our re-examination of the type specimens of *Barathricolarimensis* does not provide sufficient support for inclusion of *Muceddinamultispinosa* and *Barathricolarimensis* in *Cyclopinella*. Additional data are needed to provide for the proposed taxonomic changes; here *Barathricola* and *Muceddina* are considered valid genera with clear distinctive characters separating them from other genera (see Key and Table 2). *Cyclopinellatincanbayensis* should remain in *Cyclopinella* although its distinctive characters may be significant enough to consider moving it to a new genus after revision. *Barathricola*, *Cyclopinella*, and *Muceddina* should remain in the Schminkepinellidae as was proposed by Martínez Arbizu (2006) until more data are available.

**Table 2.** Morphological differences, distributions and habitats among species of the Schminkepinellidae.

Characters\ Species	* Muceddina multispinosa *	* Cycliinella tincanbayensis *	* C. tumidula *	* Barathricola rimensis *	*B.thermophilus* sp. nov.	* Einslepinella ulrichi *	* E. mediana *	* E. alignatha *	* Schminkepinella plumifera *
♀ Caudal ramus, L/W ratio	7.6	4.0	about 4	11.0	8.9	8	–	8	15.5
Segments of ♀ antennule	15	15	12	14	14	7	8	8	8
Armature of antenna	1-1-5-7	1-1-5-6	0-1-4-7	0-1-5-7	0-1-5-7	0-1-1-5	0-1-1-6	0-1-1-6	1-9
Inner seta on basis of mandible	Present	Present	Present	Absent	Absent	Absent	Absent	Present	Absent
Armature of mandibular exopod	1-1-1-2	1-1-2	1 seta	0-0-2	0-0-2	1-1-1	1-2	1-1-1	2
Armature of mandibular endopod	3-5	2-4	4	2-4	2-4	5	4	4	4
Setae on maxillular basis	4	?	3	4	4	4	–	–	3
Setae on maxillular exopod	4	4	3	4	4	4	–	–	4
Setae on maxillular endopod	6	?	5	5	5	–	–	–	4
Setae on maxilliped segments	5-2-1-1-1-1-4	4-2-1-1-1-1-4	3-2-1-1-3	3-2-1-1-1-1-3	3-2-1-1-1-1-3	0-0-0-1+spine	0-0-0-1	1	1-1
Outer element of 3^rd^ endopodal segment of leg 1	Seta	Seta	Spine	Seta	Seta	Spine	Spine	Spine	None
Armature of 3^rd^ endopodal segment of leg 3	3 spines + 3 setae	2 spines + 4 setae	2 spines + 4 setae	3 spines + 3 setae	3 spines + 3 setae	4 spines + 2 setae	4 spines + 2 setae	–	2 spines + 2 setae
Spines on 3^rd^ exopodal segment of leg 3	4	3	4	4	4	4	4	–	4
Armature on 2^nd^ endopodal segment of leg 4	1 spine + 1 seta	2 setae	2 setae	1 spine + 1 seta	1 spine + 1 seta	1 spine + 1 seta	1 spine + 1 seta	–	1 spine + 1 seta
Armature of exopod of ♀ leg 5	I, I+1+I	II, 1+I	I, I+1+I	I, I+1+I	I, I+1+I	I, I+1+I	I, I+1+I	I, I+1+I	1+1+I
Armature of exopod of ♂ leg 5	I-1; I+1+I, 1	Unknown	As in female	I, I+1+I, 1	As in female	I-1; I+1+I	Unknown	Unknown	I, I+1+I, 1
Distributions (habitats)	Mediterranean & Atlantic (anchihaline caves)	Australia (littoral, interstitial)	Norway (shallow water)	Northeast Pacific (hydrothermal vent area)	Indian Ocean (hydrothermal vent area)	Arctic (depth 8–529 m)	Arctic (depth 156–449 m)	Arctic (depth 256 m)	Arctic (depth 3211 m)
References	Jaume & Boxshall, 1996	Karanovic, 2008	Sars, 1913	Humes, 1998 and this paper	This paper	Martínez Arbizu 2006	Martínez Arbizu 2006	Martínez Arbizu 2006	Martínez Arbizu 2006

Data here show that the sexual dimorphism in leg 3 occurs in *B.thermophilus* and *B.rimensis*. Thus, the sexually dimorphic leg 3 known from the two species living in the hydrothermal vent environment is clearly the derived character of the genus *Barathricola* as mentioned by Martínez Arbizu (2006). *Barathricolathermophilus* sp. nov. shares with *B.rimensis* the shape of the mandibular palp and a number of other characters, e.g., Humes (1999) described the mandibular exopod of *B.rimensis* as “a minute process carrying two long setae”, but his illustrations and those here for this appendage show that the exopod is indistinctly 3-segmented, with two setae on the third segment, as in *B.thermophilus* sp. nov. In addition, the two species share the identical armature formula for the antenna (0-1-5-7), the loss of an inner seta on the basis of the mandible, a two-segmented mandibular endopod bearing two and four setae on the first and second segments, respectively, and elongate caudal rami.

Although the two species of *Barathricola* are very similar to each other, they cannot be treated as conspecific due to a significant difference in leg 5 of the male. The exopod (terminal segment) of leg 5 is armed with three spines and two setae (formula I, I+1+I, 1) in *B.rimensis*, in contrast to three spines and one seta (formula I, I+1+I) in *B.thermophilus* sp. nov. lacking a seta on the inner margin of the exopod. Within the Schminkepinellidae males of six species are known, including *B.rimensis* and *B.thermophilus* sp. nov. In these species a sexual dimorphic leg 5, as in *B.rimensis*, is known in *Muceddinamultispinosa*, *Schminkepinellaplumifera*, and *Einslepinellaulrichi*. However, Sars (1913) recorded that leg 5 of male *Cyclopinellatumidula* is of exactly the same appearance as in the female. Thus, the sexual dimorphism in leg 5 appears to be a character differentiating species, but not genera, in the Schminkepinellidae. An additional morphological difference between the two species of *Barathricola* is the ratio of the length to the width of the caudal ramus is 11.0:1 in the female and 8.5:1 in the male of *B.rimensis*, which is 8.9:1 in the female and 6.1:1 in the male of *B.thermophilus* sp. nov.

#### Key to species of the family Schminkepinellidae

**Table:** 

1	Antennule of female 7 or 8-segmented; maxilliped 1 to 4-segmented	**2**
–	Antennule of female 12 to15-segmented; maxilliped 5 to7-segmented	**5**
2	Antenna 2-segmented; third endopodal segment of leg 3 armed with 2 spines and 2 setae; third endopodal segment of leg 1 without outer element	***Schminkepinellaplumifera* Martínez Arbizu, 2006**
–	Antenna 4-segmented; third endopodal segment of leg 3 armed with 4 spines and 2 setae; third endopodal segment of leg 1 with outer spine	**3 (*Einslepinella*)**
3	Mandibular basis with inner seta; maxilliped 1-segmented	***Einslepinellaalignatha* Martínez Arbizu, 2006**
–	Mandibular basis without inner seta; maxilliped 4-segmented	**4**
4	Mandibular endopod armed with 5 setae; terminal segment of maxilliped with 1 spine and 1 seta	***Einslepinellaulrichi* Martínez Arbizu, 2006**
–	Mandibular endopod armed with 4 setae; terminal segment of maxilliped with 1 seta only	***Einslepinellamediana* Martínez Arbizu, 2006**
5	Second endopodal segment of leg 4 armed with 2 setae; third endopodal segment of leg 3 armed with 2 spines and 4 setae	**6 (*Cyclopinella*)**
–	Second endopodal segment of leg 4 armed with 1 spine and 1 seta; third endopodal segment of leg 3 armed with 3 spines and 3 setae	**7**
6	Antenna with armature formula 0-1-4-7; mandibular endopod 1-segmented, with 4 setae; maxilliped 5-segmented	***Cyclopinellatumidula* Sars, 1913**
–	Antenna with armature formula 1-1-5-6; mandibular endopod 2-segmented, with 2 and 4 setae on first and second segments, respectively; maxilliped 7-segmented	***Cyclopinellatincanbayensis* Karanovic, 2008**
7	Antenna with armature formula 1-1-5-7; mandibular basis with inner seta; mandibular endopod with 3 and 5 setae on first and second segments, respectively; first segment of maxilliped with 5 setae	***Muceddinamultispinosa* Jaume & Boxshall, 1996**
–	Antenna with armature formula 0-1-5-7; mandibular basis lacking inner seta; mandibular endopod with 2 and 4 setae on first and second segments, respectively; first segment of maxilliped with 3 setae	**8 (*Barathricola***)
8	Leg 5 sexually dimorphic, with exopod bearing 3 spines + 1 seta in female and 3 spines + 2 setae in male; length/width ratio of caudal ramus 11:1 in female and 8.5:1 in male	***Barathricolarimensis* Humes, 1999**
–	Leg 5 of both sexes with exopod bearing 3 spines + 1 seta; length/width ratio of caudal ramus 8.9:1 in female and 6.1:1 in male	***Barathricolathermophilus* sp. nov.**

## Supplementary Material

XML Treatment for Barathricola

XML Treatment for Barathricola thermophilus

XML Treatment for Barathricola rimensis
